# High-Density Electrical Recording and Impedance Imaging With a Multi-Modal CMOS Multi-Electrode Array Chip

**DOI:** 10.3389/fnins.2019.00641

**Published:** 2019-06-25

**Authors:** Beatrice Miccoli, Carolina Mora Lopez, Erkuden Goikoetxea, Jan Putzeys, Makrina Sekeri, Olga Krylychkina, Shuo-Wen Chang, Andrea Firrincieli, Alexandru Andrei, Veerle Reumers, Dries Braeken

**Affiliations:** ^1^IMEC, Heverlee, Belgium; ^2^KU Leuven, Leuven, Belgium

**Keywords:** multi-modal, impedance imaging, hippocampal neurons, CMOS, high density, multielectrode array

## Abstract

Multi-electrode arrays, both active or passive, emerged as ideal technologies to unveil intricated electrophysiological dynamics of cells and tissues. Active MEAs, designed using complementary metal oxide semiconductor technology (CMOS), stand over passive devices thanks to the possibility of achieving single-cell resolution, the reduced electrode size, the reduced crosstalk and the higher functionality and portability. Nevertheless, most of the reported CMOS MEA systems mainly rely on a single operational modality, which strongly hampers the applicability range of a single device. This can be a limiting factor considering that most biological and electrophysiological dynamics are often based on the synergy of multiple and complex mechanisms acting from different angles on the same phenomena. Here, we designed a CMOS MEA chip with 16,384 titanium nitride electrodes, 6 independent operational modalities and 1,024 parallel recording channels for neuro-electrophysiological studies. Sixteen independent active areas are patterned on the chip surface forming a 4 × 4 matrix, each one including 1,024 electrodes. Electrodes of four different sizes are present on the chip surface, ranging from 2.5 × 3.5 μm^2^ up to 11 × 11.0 μm^2^, with 15 μm pitch. In this paper, we exploited the impedance monitoring and voltage recording modalities not only to monitor the growth and development of primary rat hippocampal neurons, but also to assess their electrophysiological activity over time showing a mean spike amplitude of 144.8 ± 84.6 μV. Fixed frequency (1 kHz) and high sampling rate (30 kHz) impedance measurements were used to evaluate the cellular adhesion and growth on the chip surface. Thanks to the high-density configuration of the electrodes, as well as their dimension and pitch, the chip can appreciate the evolutions of the cell culture morphology starting from the moment of the seeding up to mature culture conditions. The measurements were confirmed by fluorescent staining. The effect of the different electrode sizes on the spike amplitudes and noise were also discussed. The multi-modality of the presented CMOS MEA allows for the simultaneous assessment of different physiological properties of the cultured neurons. Therefore, it can pave the way both to answer complex fundamental neuroscience questions as well as to aid the current drug-development paradigm.

## Introduction

*In vitro* neuronal networks are used to study communication in physiological and disease states (Obeso, 2010; Gitler et al., 2017; Dawson et al., 2018). Nerve cells, derived from animal or of human origin, form densely organized cellular networks which become electrically active after few weeks in culture (Biffi et al., 2013; Seidel et al., 2017). Patch clamp is the *de facto* golden standard for the measurement of ion currents and action potentials with high temporal resolution in these cultured neural networks (Kodandaramaiah et al., 2012). However, patch clamp is time consuming, lacks spatial resolution, requires specific expertise and extensive training and is limited to a little number of cells for each trial. It is also invasive thus long-term measurements cannot be obtained. Differently, multi-electrode arrays (MEAs) require little training and offer multi-site read out of extracellular potentials of neuronal networks (Maccione et al., 2015; Obien et al., 2015; Jans et al., 2017). However, the electrical activity of cells can only be assessed after they have reached a mature stage characterized by synaptic connections (Ichikawa et al., 1993; Hormuzdi et al., 2004; Spitzer, 2006). To study cell adhesion and growth at earlier cultures stages, optical microscopy is mostly used (Delgado Ruz and Schultz, 2014). This approach often requires invasive protocols which may not only influence the culture itself, but also require end-point measurements. An alternative technique to study cell adhesion and growth is the electrochemical impedance spectroscopy (EIS) (Daniels and Pourmand, 2007). In EIS, a small alternating current is applied to a working electrode and the resulting voltage drop across the sample is measured at an opposite reference electrode. EIS does not require labels, it is non-invasive for cells, i.e., allowing long-term analysis, and it can be performed with high sampling rates at several frequencies of interest to assess different physiological phenomena (Xu et al., 2016). In addition to voltage recording, the electrodes on the MEA chips can also be used for EIS (Manickam et al., 2010; Chen et al., 2012; Pui et al., 2013; Goikoetxea et al., 2018; Park et al., 2018b; Viswam et al., 2018a).

Most MEAs consist of maximally a few hundred of electrodes embedded in a glass or silicon substrate, also referred as passive MEAs (Zitzmann et al., 2017). In this case, the signal amplification, filtering and signal actuation are done off-chip. This not only limits the portability, i.e., applicability of the device, but it also requires external cable connections that contribute to increase the total noise level and crosstalk of the system (Huys et al., 2012). Differently, active MEAs integrate electronic components such as filters, switches, signal generators, amplifiers and analog-to-digital converters directly underneath the electrodes surface, thus offering superior sensing and actuation capabilities (Hierlemann et al., 2011; Obien et al., 2015). This is why, they also provide the spatial resolution required to investigate in details single-cell behavior within large neuronal networks (Müller et al., 2015). Nevertheless, the majority of the reported CMOS MEA mainly rely on a single operational modality, therefore strongly hampering the applicability range of a single device (Chi et al., 2015). This can be a limiting factor considering that the majority of biological and electro-physiological dynamics are often based on the synergy of multiple and complex mechanisms acting from different angles on the same phenomena (Chi et al., 2015; Park et al., 2018b). This is particularly relevant in the field of drug development and testing in which multi-parameter cellular phenotypic profiling can be a promising approach to have multiplexed measurements of the numerous cellular pathways involved (Feng et al., 2009). For example, having the possibility to perform electrical measurements both at the cellular and network level is fundamental to deeply unveil how individual cells can impact the overall neuronal network and vice versa (Müller et al., 2015). This is translated in specific technological requirements and challenges that have to be faced to develop cutting-edge MEAs to solve advance neuroscience problems.

Here, we present the investigation of *in vitro* neuronal hippocampal networks with a multi-modal CMOS MEA chip. The chip features 16,384 titanium nitride (TiN) electrodes arranged in 16 different active areas and with an electrode pitch of 15 μm (Lopez et al., 2018). Each active area includes 1,024 electrodes grouped in 256 pixels (four electrodes per pixel). The six modalities include both voltage and current stimulation, intracellular and extracellular recording, along with impedance measurements. Two impedance modalities are present to perform both impedance monitoring at a fixed frequency (1 and 10 kHz), and impedance spectroscopy in the range between 10 Hz and 1 MHz. The here presented CMOS MEA chip includes the largest number of operating modalities if compared with other MEA chips present in the literature and commercially available (Lopez et al., 2018). Moreover, the electrode pitch is only 15 μm thus allowing for higher electrode densities and single cell resolution if compared with other reported devices (Abbott et al., 2017; Park et al., 2018a; Tsai et al., 2018). Besides this, the fabrication process, including the electrode, is fully CMOS compatible, and thus allows for reproducible manufacturing conditions.

In this paper, we exploit the impedance monitoring and voltage recording modalities not only to monitor the growth and development of primary rat hippocampal neurons, but also to assess their electrophysiological activity. Fixed frequency (1 kHz) at high sampling rate (30 kHz) impedance measurements are used to evaluate the cellular adhesion and growth on the chip surface. The cell culture was monitored starting from a few hours after seeding (4.5 h) and then over several days (8 days *in vitro*, DIV) using the on-chip fixed-frequency impedance circuits. Spontaneous electrical activity under the form of single unit and synchronized network activity was recorded at 15 and 43 DIV using the voltage recording circuits present in the same pixels. We also assessed the noise and signal amplitude from neuronal recordings for the four electrode sizes on the chip. Cell live staining was used as a comparison for the electrical imaging obtained by the impedance measurements on-chip. The flexibility in the electrode selection allowed for a multi-functional readout of the same cells along a large surface area at high spatial resolution.

## Materials and Methods

### Primary Hippocampal Neurons (PHN) Culture on the Multimodal CMOS MEA Chip

To perform the cell culture on the multimodal CMOS MEA chip, a glass ring (height 0.8 cm and diameter 3.4 cm) was glued on the printed circuit board (PCB) around the CMOS chip using biocompatible epoxy. The CMOS MEA chips were then sterilized and coated as follows [the coating procedure was adapted from Amin et al. (2016)]. After wiping the entire PCB surface with 70 % ethanol, the active area of the CMOS chip was sterilized by filling the glass ring with 70 % ethanol for 15 min. After 3 rinsing steps with sterile highly purified water (HPW) the chip was coated overnight at 37°C using 50 μg/mL poly-dl-ornithine (PDLO, Sigma) dissolved in a borate buffer solution. This step was necessary not only to promote the cellular adhesion but also to increase the hydrophilicity of the chip surface. After 24 h, the chip surface was rinsed abundantly with sterile HPW and let dry for about 2–3 h prior the culture.

All the experiments involving live animals were executed according to guidelines approved by the local university animal ethics committee and compliant with the European Communities Council Directive of November 24, 1986 (86/609/EEC).

Gestating Wistar rats (E19) were euthanized using carbon dioxide and the embryos were moved in a Ca^2+^ and Mg^2+^ free Hank’s balanced salt solution (HBSS, Gibco^®^). After dissection, the hippocampi were enzymatically dissociated in 5 mL of 0.05% trypsin-ethylenediaminetetraacetic (EDTA, Gibco^®^) at 37°C for 15 min under gentle agitation. Further dissociation was achieved by pipetting with a fire-polished Pasteur pipette after re-suspension of the supernatants in the plating medium composed by Neurobasal^®^ medium electro with B-27^®^ supplement (NB, Gibco^®^) and 15 % of fetal bovine serum (FBS, Gibco^®^). After counting, the cells were plated on the CMOS MEA chip using a density of 75,000 cells/cm^2^. In case of low concentration, the cells were centrifuged for 5 min and resuspended in the desired amount of medium. After 24 h the medium was replaced with culture medium including only NB. Up to 3 DIV also L-glutamic-acid (0.25 μM, Sigma-Aldrich) was added to the culture medium. The isolate neurons were then cultured for more than 30 days. After culturing, the chips were cleaned with a 1% Terg-A-zyme^®^ solution (Alconox^®^, Sigma-Aldrich) for 5–10 min at 37°C that will enzymatically digest the cells and then washed 4 times with HPW. The chips were then inspected with optical microscopy to assure the success of the cleaning procedure. In case of residues present on the chip surface, the process was repeated. A similar cleaning protocol was also reported in Welkenhuysen et al. (2016) and it allowed to re-use the chips several times, if wanted.

### Electrical Measurements With the CMOS Chip

**FIGURE 1:**
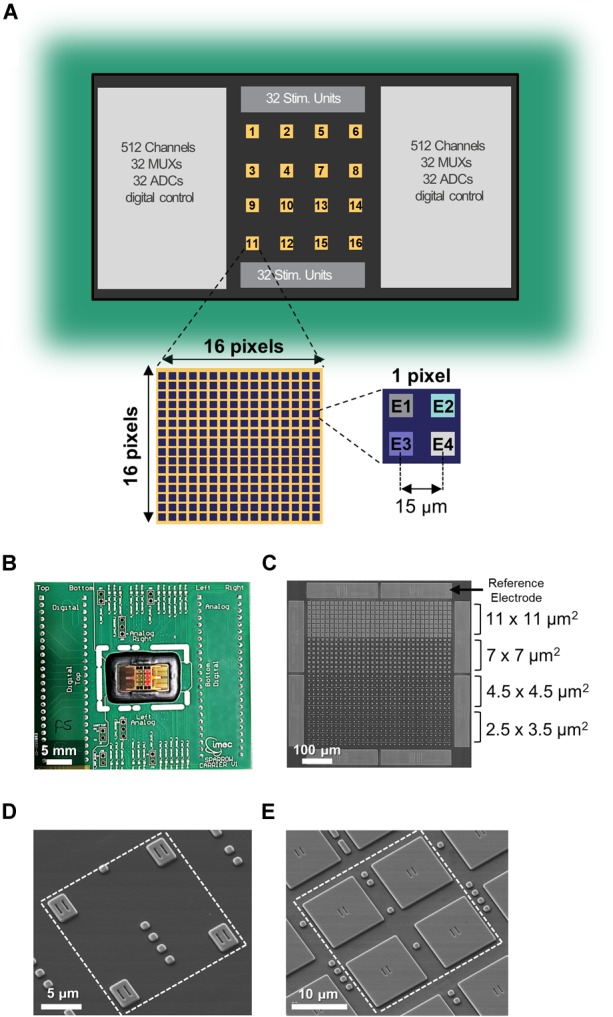
Multi-modal CMOS MEA overview. **(A)** Schematic of the overall CMOS MEA chip with zoom on a single active area and on a single pixel. **(B)** Picture of the CMOS MEA chip wirebonded on the carrier PCB. **(C)** SEM image of one active area featuring the four different electrode sizes and the eight reference electrodes. **(D,E)** SEM images of a single pixel containing electrodes of sizes 2.5 × 3.5 μm^2^ **(D)** or 11 × 11 μm^2^ **(E)**.

The CMOS MEA chip was fabricated using a standard 0.13 μm CMOS process with a 6-metal-layer aluminum back-end-of-line (BEOL) stack as described in Lopez et al. (2018) and (Jun et al., 2017). An array of 16,384 TiN electrodes was then patterned on the chip surface using reactive ion etching (RIE) (Lopez et al., 2018). The electrodes are divided in 16 active areas and connected to 4096 pixels (256 pixels/active area). As it will be described in detail in Section “Multimodal CMOS MEA Chip,” 4 electrodes are present in each pixel, selectable by means of a 4-to-1 multiplexer. A maximum of 1024 pixels can be then connected to the 1024 recording channels then time-multiplexed at 30 kS/s. The usual recording configuration used in the present paper consisted in simultaneously enabling all the 256 pixels in 4 active areas (256 × 4 = 1024 total pixels, areas 1–4 according to the schematic in Figure 1A). Therefore, simultaneously recording from all the 4 electrode sizes. During each measurement, the pixels were connected to 1 of the 4 electrodes resulting in a 1024-electrodes recording. To record from all the 4 electrodes in a pixel, four sequential recordings were performed switching between the 4 electrodes. An AC-coupled source-follower is also present in every pixel for low-impedance output. The 1024 recording channels have programmable gain ranging between 2 and 3000 V/V and a bandwidth selectable between 0.5–10000 Hz or 0.3–10 kHz. A reconfigurable instrumentation amplifier allows to switch between 4 different operating modalities in the same recording channel (i.e., extracellular recording, intracellular recording, impedance monitoring and impedance spectroscopy). In this work we focused on 2 of these 4 modalities: extracellular recording and impedance monitoring. In case of extracellular recording, the cellular signal is acquired by connecting the channel to a selected pixel. Instead, during impedance monitoring, square-wave current sources, also present in the recording channel, are directly connected to one electrode [see Supplementary Figure S1 and (Lopez et al., 2018)]. More detailed information about the chip architecture and design can be found in Lopez et al. (2018).

The CMOS MEA chips were wirebonded and packaged onto individual PCBs and plugged into a custom-designed bench-test setup which allowed to supply the necessary voltages as previously reported (Lopez et al., 2018). The whole setup was battery-powered and a digital I/O card (PXI 6544, National Instruments) was employed to configure and acquire data from the chip.

As previously mentioned, we focused on 2 of the 6 modalities present in the chip: impedance monitoring and voltage recording. The impedance monitoring mode operates by applying a on-chip generated square-wave current excitation signal (1 kHz, 0.5 nA) and then measuring the resulting AC voltage at the corresponding channel. The impedance monitoring circuit was also used to acquire the baseline impedance of the electrodes in a standard saline solution (phosphate buffer solution, PBS, Gibco^®^). Thanks to the low amplitude of the current applied to perform the impedance measurement, i.e., low voltages, there was no alteration of the physiological properties of the cells, or of their *trans-*membrane currents (Peters et al., 2015; Lopez et al., 2018).

During voltage recording, the extracellular signal is recorded by an instrumentation amplifier with programable gain. The channel gain was set to 50 or to 500 for impedance or extracellular recordings, respectively. Moreover, during extracellular recording the channel bandwidth was programmed to be between 0.3–10 kHz. All the voltage measurements were performed using the on-chip reference electrodes (see Section “Multimodal CMOS MEA Chip”). All the electrical measurements on the primary hippocampal neurons were performed at 37°C.

### Fluorescent Imaging

For the immunostaining experiments, primary hippocampal neurons were fixated in a 4% formaldehyde solution for 10 min (Thermo Fisher Scientific). Then, they were permeabilized using a 0.2% solution of Triton X (Sigma-Aldrich) in PBS for 5 min. Next, a 20% goat serum (Sigma-Aldrich) solution in 0.2% Triton X was used for blocking (20 min). To stain the microtubule associated protein 2 (MAP2) in neurons, the chicken polyclonal anti-MAP2 antibody (ab92434, abcam) and the goat anti-chicken IgY Alexa Fluor 488 (A11039, Invitrogen) were selected as primary and secondary antibody, respectively. Instead, to map the glial fibrillary acidic protein (GFAP) in glia cells, the polyclonal rabbit anti-GFAP (Z0334, Dako) and goat anti-rabbit IgG Alexa Fluor 568 (A11036, Invitrogen) were selected as primary and secondary antibodies, respectively. After washing the blocking solution with PBS, the samples were incubated with the primary antibodies overnight at 4°C. Afterward, the secondary antibodies were incubated for 1 h at room temperature. Moreover, 4′,6-Diamidino-2-Phenylindole, Dihydrochloride (DAPI, D1306 Invitrogen) was also added to the secondary antibody solution for nuclei visualization.

Live cell imaging was also used as a comparison to the electrical imaging. In this case, neurons were loaded with Calcein green AM (0.1 μL/mL, Invitrogen, 1-h incubation at 37°C). All the fluorescent images were taken using a confocal microscope (Zeiss Laser Scanning Microscope, LSM 780).

### Scanning Electron Microscopy Characterization

The Scanning Electron Microscopy (SEM) characterizations were performed using a FEI Nova 200 NanoSEM operating at 7 kV and equipped with an Everhart-Thornley detector (ETD). Unpackaged dummy chips were used for the characterization of the CMOS MEA electrodes.

Neuronal SEM samples (3 DIV) were prepared as described in Section “Primary Hippocampal Neurons (PHN) Culture on the Multimodal CMOS MEA Chip” and seeded on dummy MEA chips at 25,000 cells/cm^2^. They were then fixed with a 2% glutaraldehyde solution and put through dehydration steps with increasing concentrations of ethanol. After that, they were chemically dried using hexamethyldisilazane (HMDS, Sigma-Aldrich).

Before imaging, all the samples were coated with 3 nm of platinum (Pt) and they were then fixed on the holder by conductive carbon tape.

### Data Analysis

All the data were analyzed using the Matlab^®^ software. During impedance measurements, in order to compensate for the intrinsic impedance differences between multiple electrodes and multiple chips, the data were normalized as followed. At first, for each electrode, the impedance data recorded from the cell culture were normalized to the average baseline values recorded in PBS (eq1). Later, the data were rescaled between 0 and 1, with 0 representing the minimum value and 1 the maximum value of the entire dataset. More specifically, 0 and 1 correspond to effective impedance increases of 11.3 and 92.5%, respectively. Outliers were also removed from the analysis.

(1) $$\Delta Z=\frac{Z_{cell}-Z_{PBS,mean}}{Z_{PBS,mean}}$$

The analysis of the voltage recording data was performed by a custom-developed script adapted from the work reported in Quian Quiroga and Nadasdy (2004). Briefly, the raw data were filtered using a Butterworth filter of order four in the frequency range between 500 and 5000 Hz. The selected range is aligned to what is commonly reported in the literature to filter out the slow local field potential oscillations (LFP) and focus on the extracellular action potentials (EAPs) (Quian Quiroga and Nadasdy, 2004; Obien et al., 2015; Jun et al., 2017). To eliminate measurements artifacts or the contribution of faulty channels/electrodes from the measurements, the data of each channel have been individually assessed using an automated algorithm based on the root mean square (RMS) noise behavior. The spike detection was performed using amplitude thresholding. More in details, an automatic threshold (Thr) was defined according to the following equation described in Quian Quiroga and Nadasdy (2004):

(2) $$Thr=6\cdot \sigma_{n}\sigma_{n}=median\left\{\frac{\left|x\right|}{0.6745}\right\}$$

Where *x* is the filtered signal (recorded from one electrode) and σ_n_ represents an estimate of the standard deviation of the noise in background (median absolute deviation, MAD). As described in Quian Quiroga and Nadasdy (2004), by selecting the median to estimate the noise the interference of the spikes in the signal is strongly reduced if compared to the direct standard deviation calculation. Selecting the optimal threshold is always a compromise between loosing the information of spikes of smaller amplitudes (threshold too high) and false positive signals (threshold too low). In our work, we selected a threshold equal to six times the MAD. After the detection of the spikes both the spike time and 60 data samples, corresponding to ∼ 2 ms, were saved for each detected signal for later analysis.

In order to evaluate the quality of the measured extracellular spikes, the maximum signal-to-noise ratio (SNR) was calculated by dividing the maximum spike amplitude detected in each channel by the MAD for each recorded channel (i.e., the total noise). Spike sorting was not performed since in this work we were mostly interested in the overall network behavior.

## Results and Discussion

### Multimodal CMOS MEA Chip

The multimodal 0.13-μm CMOS MEA chip features 16,384 electrodes arranged in 4096 pixels, 1024 simultaneous readout channels, 64 MUXs, 64 stimulation units, and 64 ADCs (Figure 1A). Figure 1B shows the chip packaged onto a carrier printed circuit board with wirebonds covered by epoxy for electrical isolation [as described in Section “Primary Hippocampal Neurons (PHN) Culture on the Multimodal CMOS MEA Chip”]. The 16 separated “active areas” on the chip surface can be simultaneously or independently accessed, and each one consists of 256 pixels in a 16 × 16 matrix configuration (Figure 1A). Each pixel contains four electrodes (Figure 1A) therefore resulting in 1024 electrodes per active area (Figure 1C) with an electrode pitch of 15 μm. Each pixel contains a 4-to-1 multiplexer to be connected to one of the four electrodes [see Supplementary Figure S1 and (Lopez et al., 2018)]. Each of the 16 active areas also contains eight additional pixels connected to the eight integrated large TiN reference electrodes (50 × 235 μm^2^) surrounding each area (Figure 1C).

TiN electrodes are post-processed in-house on the CMOS chip with a six-metal-layer aluminum BEOL process as described in Section “Electrical Measurements With the CMOS Chip.” The chip was populated with four different electrode sizes in order to assess the influence of electrode size on measurement signal amplitude and noise. The four different electrode sizes can be appreciated in Figure 1C and are, from large to small, 11 × 11 μm^2^, 7 × 7 μm^2^, 4.5 × 4.5 μm^2^, and 2.5 × 3.5 μm^2^. Figure 1D,E display a representative SEM images of 1 pixel featuring electrodes of 2.5 × 3.5 μm^2^ and 11 × 11 μm^2^, respectively (see Supplementary Figure S2 for additional SEM images of the other two electrode sizes).

### Electrical Imaging Using On-Chip Fixed-Frequency Impedance

**FIGURE 2:**
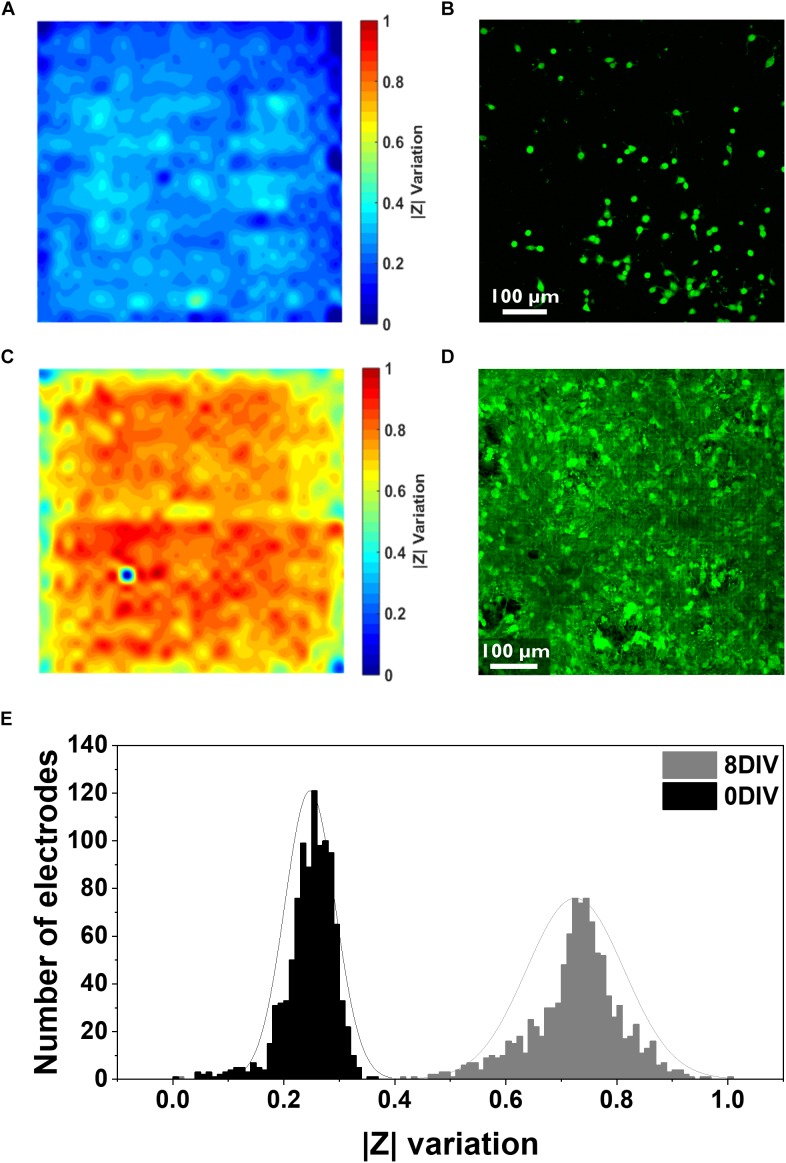
Electrical and confocal imaging of primary hippocampal cultures at 0 and 8 DIV. **(A,B)** Electrical impedance map and confocal image of the cell culture stained with Calcein AM 4.5 h after seeding. **(C,D)** Electrical impedance map and confocal image of an 8 DIV confluent culture stained with Calcein AM. Both electrical and confocal images correspond to the same chip surface area (one active area, i.e., 1024 electrodes, 2500 μm^2^). **(E)** Histogram illustrating the distribution of the relative impedance variation recorded by 1024 electrodes for the 0 DIV (black) and 8 DIV (gray) cultures.

Non-invasive, label-free imaging of cell adhesion and growth is of interest for virtually every cell culture application (Asphahani and Zhang, 2007; Heileman et al., 2013; Xu et al., 2016). There are numerous spatial features such as pre- and post-synaptic neuron location, phenotypic features or different groups of cellular morphologies that are crucial to characterize a neural circuit and that are often put in the background compared to extracellular recordings (Delgado Ruz and Schultz, 2014). Nevertheless, they are crucial for improving and expanding our current knowledge about the functionality and potential of *in vitro* neural networks, especially for drug screening purposes (Feng et al., 2009; Delgado Ruz and Schultz, 2014). In addition to its importance for assessing multiple phenotypic cues, as previously mentioned, impedance monitoring is also key for other studies such as cell viability, proliferation and adhesion which are necessary for developing innovative strategies against multiple diseases including cancer and wound healing (Park et al., 2018b). Our first aim was to use the impedance monitoring feature of our CMOS MEA platform to distinguish between a freshly seeded and a confluent culture. To study cell adhesion right after seeding, we measured the impedance magnitude of the cell culture after 4.5 h from seeding using the 1 kHz impedance monitoring setting of the MEA chip. Figure 2A shows the impedance recording of 1 complete active area on the chip (all 1024 electrodes). As previously described in Section “Data Analysis,” the impedance is normalized to the mean values calculated in PBS in order to account for intrinsic differences between chips and to facilitate data interpretation.

Cells were then stained with the live staining marker Calcein Green-AM to compare it with the impedance mapping (Figure 2B). This dye binds free Ca^2+^ present in the cell cytosol. As it can be seen from the comparison, there is an overlap of cell presence monitored by both techniques, although the impedance data seems to demonstrate a more detailed view of the cell culture. This can be explained by the fact that the Calcein staining underestimates the complete cell contour, especially very thinly spread cell parts. Besides this, the sensitivity of the Ca^2+^ indicator depends on the amount of free Ca^2+^ in the cell and the sensitivity of the microscope technique, while the impedance signal is directly related to the current path which gets disturbed by the presence of cells. More specifically, a cell covering an electrode acts like a barrier for the current path, and therefore the overall recorded impedance increases. These variations in impedance depend on the cell-electrode distance, which is linked to cell adherence (Giaever and Keese, 1991). Apart from the advantages previously mentioned, there is also a clear difference in the time needed to obtain the results: while the impedance monitoring only takes about 2 min, taking a confocal image of the entire chip surface takes about 20–30 min. Instead, considering a confluent culture (8 DIV), a clear complete coverage of the chip surface can be seen from both techniques (Figure 2C,D). The histogram with the distribution of the relative changes in impedance amplitude for both the initial and the 8 DIV cultures shows that the mean variation in the first hours of the culture is considerably lower (0.24 ± 0.04) compared to 8 DIV (0.72 ± 0.08) (Figure 2E). As extensively reported in the literature, high relative variations in impedance translate into strongly attached cells since, in this case, most of the current is blocked (Qiu et al., 2008; Chen et al., 2012). Further, in the initial culture up to 55% of the electrodes show no relative changes in impedance compared to the baseline measurement performed in PBS. This result indicates a low degree of cell adhesion as 4.5 h are not enough for cells to completely adhere to the chip surface. In agreement with the impedance data, the confocal microscopy picture of the initial culture in Figure 2B depicts how cells are still in the process of adhering or have not firmly adhered yet. Instead, when the culture reaches a confluent stage, a gaussian-like adhesion profile can be observed related to the higher degree of cellular adhesion on the chip surface. Similarly, the confocal micrograph corroborates the impedance data as it shows a fully confluent cell culture. The impedance and confocal imaging measurements were performed in order to guarantee an adequate temporal correspondence between the two experiments.

### High Density Recording of Single Spikes and Synchronized Activity

Primary rat hippocampal neurons are an excellent model system to study neuronal communication (Beaudoin et al., 2012; Dawson et al., 2018) so this culture model was chosen to validate the voltage recording feature of the CMOS chip. When hippocampal neurons are kept in culture for a sufficiently long time (minimum 2 weeks), spontaneous electrical activity can be recorded (Mazzoni et al., 2007; Charlesworth et al., 2011). From this moment, synchronized network-wide activity can be measured as groups of spikes in a wide range of frequencies and inter-burst intervals (Mazzoni et al., 2007; Gandolfo et al., 2010; Kim et al., 2014).

**FIGURE 3:**
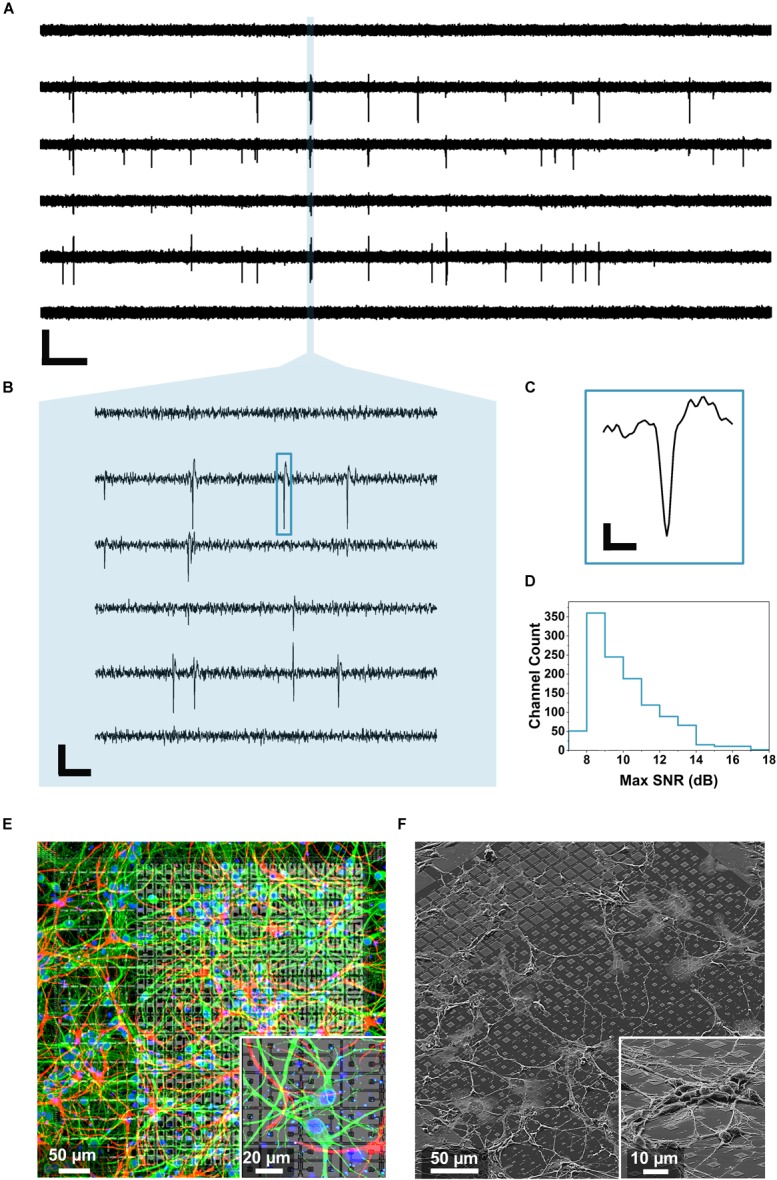
Neuronal voltage recordings and microscopy. **(A)** Raw traces of the spontaneous electrical activity recorded from primary hippocampal neurons at 15 DIV on 6 of the 1024 electrodes for 30 s (scale bar: 400 μV vertical, 2 s horizontal). **(B)** Zoom over 100 ms of the recorded spontaneous activity unveiling individual spikes (scale bar: 200 μV vertical, 10 ms horizontal). **(C)** Profile of an individual spike corresponding to the blue rectangle in **B** (scale bar: 100 μV vertical, 0.5 ms horizontal). **(D)** Maximum SNR calculated from 1024 electrodes during five sequential recordings according to the method described in Section “Data Analysis” and showing an average SNR of 10.1 ± 1.9 dB. **(E)** Immunostaining of a 14 DIV primary hippocampal neuron culture on a single active area of the chip and on a zoomed area (inset). The MAP2 immunostaining mapping neurons can be observed in green, the GFAP mapping the glia present in the culture is reported in red while the nuclei are shown in blue (DAPI). **(F)** SEM images of a 3 DIV primary hippocampal neuron culture on a single active area and on a zoomed region (inset) of the CMOS chip.

To record these features, hippocampal neurons were cultured on the chip for minimally 15 days. Then, their spontaneous activity was recorded by 1024 electrodes at the same time in 4 active areas (1 electrode per pixel, active areas 1–4). As explained in Section “Electrical Measurements With the CMOS Chip,” four sequential recordings were performed in order to record the activity from all the 4 electrodes on the pixels. Figure 3A shows representative raw voltage recording traces from 6 of the 1024 electrodes used for one measurement over a time period of 30 s. The data were bandpass filtered (500 Hz–5 kHz) and a threshold of 6 σ_noise_ was applied for the spike detection (see details section “Data Analysis”). At 15 DIV the neuronal network is functional but still growing and expanding on the MEA surface therefore resulting in frequent and short synchronized activity events, as reported previously (Mescola et al., 2016). In order to resolve individual spikes, shorter time scales need to be considered, as shown in the zooms of Figure 3B (100 ms) and Figure 3C (2 ms). Further, we determined the SNR considering all the 1024 recorded electrodes in five sequential measurements (procedure described in section “Data Analysis”). As outlined in Figure 3D, an average SNR of 10.1 ± 1.9 dB was calculated showing how most of the traces display signals that can be well distinguished from the noise. To visually identify neuronal viability and outgrowth we then stained 2 weeks-old neuronal cultures with fluorescent markers for neurons (MAP2, green), cell nuclei (DAPI, blue) and glial cells (GFAP, red) as shown in Figure 3E and inset (see section “Fluorescent Imaging” for the staining details). As can be observed from Figure 3D,E, after 14 DIV, the cells formed an intricated network spreading over the entire active area surface. In particular, the cell culture protocol (specifically the coating and seeding density) was optimized to limit the clustering phenomena that can be observed in aged neuronal cultures and which may interfere with the culture spatial arrangement (Zeng et al., 2007). We also preformed SEM on cultured neurons on chip (3DIV), following the procedure described in Section “Scanning Electron Microscopy Characterization.” Figure 3F shows extended neuronal networks over an individual active area and with larger magnification on a smaller region (inset). After culturing, the chips could be cleaned using the protocol described in Section “Primary Hippocampal Neurons (PHN) Culture on the Multimodal CMOS MEA Chip,” and they could be re-used several times (up to three to five times), if desired (Welkenhuysen et al., 2016).

**FIGURE 4:**
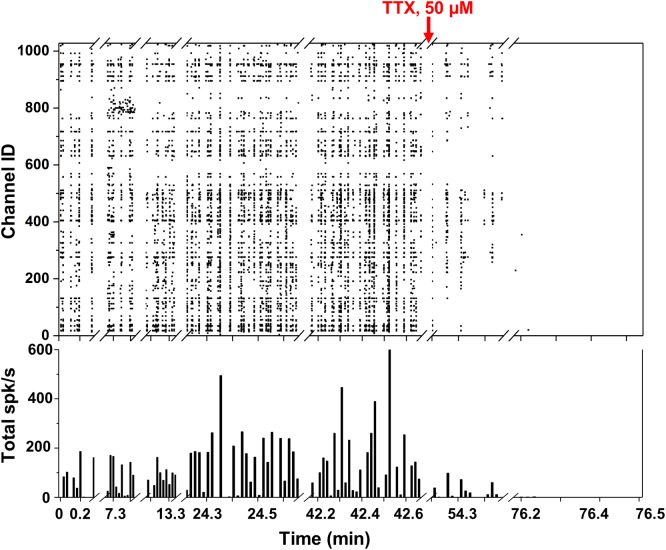
Spontaneous network activity and TTX experiment. (**Top**) Raster plot showing the network spiking activity (*y* axis) of 1024 electrodes in time (*x* axis) over more than 1 h. Each black square corresponds to an identified spike. (**Bottom**) Total spikes/s bar plot with 1 bin = 1 s.

We took several measurements over more than 1 h from the neuronal culture and represented the overall network behavior into a single raster plot (Figure 4). Spikes detected with the thresholding algorithm described in section “Data Analysis” (>6 σ_noise_) are represented as black squares displaying the neuronal network synchronicity at 15 DIV. The network synchronous behavior can be also identified in the histogram at the bottom of Figure 4 showing, for each second (1 bin = 1 s), the total number of detected spikes per second (spikes/s). This activity could be abolished by adding the Na^+^ blocker TTX (50 μM), as displayed by the diminishing detection of spikes after addition of this compound (red arrow in Figure 4). The non-uniform frequency of the synchronized activity was probably due to the different and dynamic transitions of the synchronized activity present in neural networks (Kim et al., 2014). The average maximum spike amplitude was 144.8 ± 84.6 μV, which is consistent with what reported in the literature (Maccione et al., 2013).

### Influence of Electrode Size on Signal Noise and Amplitude

As the CMOS chip is populated with thousands of electrodes, and those electrodes are divided in four different electrode size groups, we can investigate the influence of electrode size on signal strength and noise behavior (see section “Data Analysis” for more details about the used methods). All the neuronal data reported in this section were acquired by enabling 1024 electrodes in 4 active areas (areas 1–4) on a 15 DIV neuronal culture (*n* = 5 measurements). When recording neural signal using planar electrodes *in vitro*, the quality of the neural recording (e.g., SNR) will depend on several factors: (i) the electrode size, (ii) the electrode impedance and the input impedance of the recording amplifier, (iii) the electrode noise and the noise of the recording amplifier, and (iv) the distance and alignment between the neuron and the electrode. These factors contribute to different signal-degradation effects that are explained as follows.

The total noise affecting neural recording has two components: (i) the thermal noise generated by the electrode-cell interface (V_n-elec_) and (ii) the noise of the readout electronics (V_n-amp_). The total noise (V_n-total_) can be calculated as:

(3) $$V_{n-total}=\sqrt{V_{n-elec}^{2}+V_{n-amp}}$$

**FIGURE 5:**
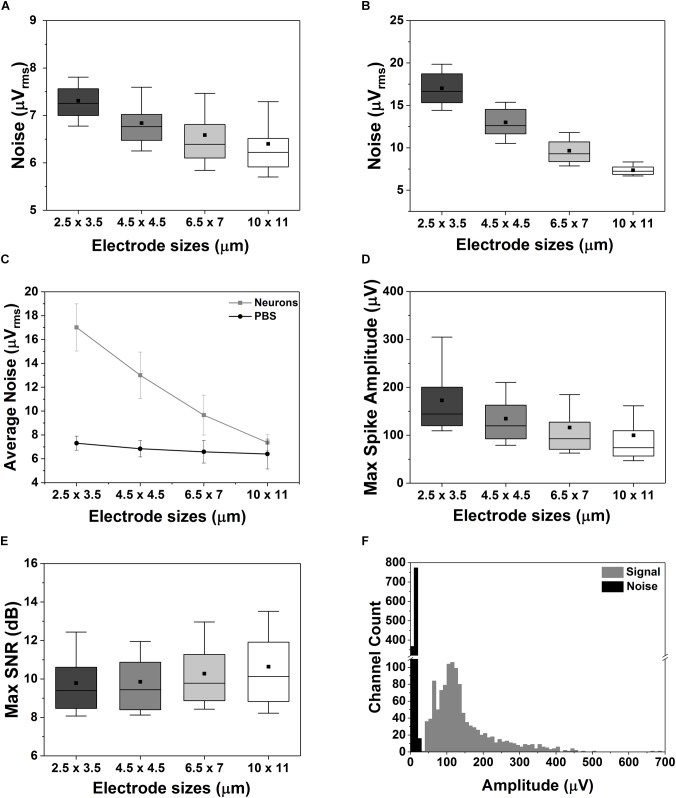
Effect of electrode sizes on noise, maximum spike amplitude and SNR. **(A–C)** Box plot of the electrode-size dependency of the noise measured in saline solution **(A)** or in case of a 15 DIV neuronal culture grown on the CMOS MEA chip **(B)**. Electrode size dependency of the average noise behavior between PBS and neuronal cultures **(C)**. **(D,E)** Box plot of the maximum spike amplitude **(D)** and of the maximum SNR **(E)** for different electrode sizes. The data were acquired on a 15 DIV neuronal culture over in *n* = 5 measurements. **(F)** Distribution of the amplitude of the signal and of the noise recorded from 1024 electrodes in *n* = 5 measurements. In all the box plots in this figure the outliers were removed, the horizontal line represents the median, the black square represents the mean, the box boundaries the 25^th^ and 75^th^ percentile while the whiskers represent the 10^th^ and 90^th^ percentile.

The noise generated by the electrode-cell (or electrode-electrolyte) interface mostly depends on the electrode area, the double-layer capacitance formed at the interface (i.e., electrode impedance) and the resistivity of the saline solution, medium or cell-membrane (Yang et al., 2010; Lopez et al., 2012; Viswam et al., 2018b). We have measured the total noise for the different electrode sizes in saline (PBS solution), with and without the presence of cells. Figure 5A shows that the total noise, V_n-total_, measured in saline is dominated by the electronics noise [∼7.5 μV_rms_ in a 300 Hz to 10 kHz bandwidth as reported in Lopez et al. (2018)] Specifically, the measured V_n-total_ in saline varies from 6.4 ± 1.3 μV_rms_ up to 7.3 ± 0.6 μV_rms_ for the largest (11 × 11 μm^2^) and the smallest (2.5 × 3.5 μm^2^) electrodes, respectively. A logical area dependency can be observed related to the higher impedance of small electrodes (see Supplementary Figure S3), as also reported in the literature (Obien et al., 2015; Viswam et al., 2018b). Nevertheless, thanks to the low impedance of the TiN electrode material, the reduction in electrode size does not impact significantly the quality of the recordings (Jun et al., 2017). Similar conclusions were made by Viswam et al. (2018b), whose work demonstrates that by electroplating their electrodes to achieve low impedance, it is possible to achieve good-quality recordings *in vitro* even with electrode diameters of less than 5 μm. In the presence of a neuronal culture (15 DIV, Figure 5B), the V_n-total_ of large electrodes is still dominated by the electronics noise since it is plausible to assume that on average the electrode is not totally covered by cells, even in mature cultures. But, when a small electrode (e.g., equal or smaller than the cell body, as visible from Figure 3E,F) is fully or mostly covered by a cell, the cell-membrane impedance will play an important role in the total noise as observed in Figure 5C, therefore escalating the area dependency. In fact, uncovered (parts of) electrodes display a lower impedance and thus lower noise behavior.

Next, we compared the maximum spike amplitudes recorded for each type of electrode (Figure 5D), and an opposite trend was observed: smaller electrodes show higher amplitudes (172.9 ± 71.6 μV and 99.9 ± 70.1 μV for the smallest and largest electrodes, respectively). This is related to the dependency of the recorded neural signal amplitude on (i) the distance between the electrode and the neural source and (ii) the electrode area. The signal amplitude can be significantly reduced due to spatial-averaging effects across the recording area of the electrodes. This spatial averaging has been studied and reported in several publications (Canakci et al., 2017; Hill et al., 2018; Viswam et al., 2018b). The work of Viswam et al. (2018b) shows that the signal from a very close source can have up to 25 % attenuation when measured with an 86-μm^2^ electrode as compared to an 11-μm^2^ electrode. This effect was also observed in our measurements as shown in Figure 5D. In our case, a signal attenuation of ∼42 ± 1.4% was observed for the largest electrodes. This means that the reduction in electrode area does not degrade significantly the maximum SNR of the neural signals recorded with planar electrodes. As shown in Figure 5E, only small to no SNR degradation is observed depending on the electrode size and the alignment of the neural source. Finally, we compared the distribution of the maximum spike amplitudes recorded by all the electrodes with the noise distribution (Figure 5F) resulting in a clear distinction between neuronal signals and noise.

### Combined High-Density Electrical Recording and Impedance Imaging

**FIGURE 6:**
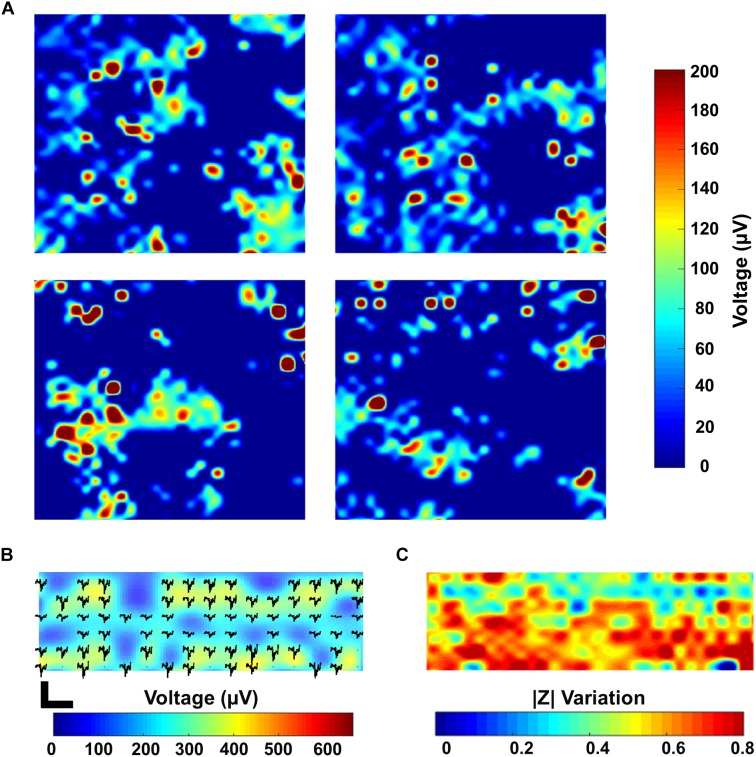
Combined electrical recording and impedance imaging. **(A)** Spike maps showing the maximum amplitude recorded in four active areas (active areas 1–4) on a 15 DIV PHN neuronal culture. Each individual map corresponds to a 500 × 500 μm^2^ active area on the chip. The map includes the measurement over all the four electrodes present in a pixel for all the 1024 pixels. This results in a total of 4096 electrodes measured in groups of 1024 (one electrode measurement per pixel). **(B,C)** Spike map and electrical impedance image of a 43 DIV PHN neuronal culture zoomed in an area corresponding to a 250 × 500 μm^2^ surface and 512 electrodes.

The simultaneous monitoring of the electrophysiological properties of cells together with spatial imaging is crucial for electrogenic cells like neurons or cardiomyocytes (Delgado Ruz and Schultz, 2014; Park et al., 2018b). Thanks to the high temporal resolution of our CMOS MEA (0.1 ms in 1 period at 1 Hz), we are able to monitor fast cellular dynamics related e.g., to the modulation of the activity of ions channels (Weyer et al., 2016), cell contractility (Lopez et al., 2018) or even the activation of G-protein coupled receptors (Zitzmann et al., 2017). Moreover, the ability to record from numerous electrodes at once allows for high-density neuronal network readout (Franke et al., 2012; Müller et al., 2015). Figure 6A shows a snapshot of the overall electrical activity recorded by sequentially enabling each of the four electrodes in every pixel using all the 1024 recording channels on a 15 DIV PHN neuronal culture. This leads to the simultaneous activity recording in 4 neighboring active areas, with the information gathered from 4096 electrodes. The chip electrode selection scheme makes it possible to record from all the 1024 electrodes in a specific active area, or to select a combination of electrodes in each of the 16 different active areas to increase the spatial resolution. Further, the different modalities are present in each pixel, which enables a fully flexible readout choice for the user: for voltage recording, the gain of each channel can be controlled individually, with a larger variation in gains to address a large variety of cell and tissue types. For impedance imaging (with a sampling rate of 30 kHz), data can be acquired at 1 or 10 kHz. Both functionalities can also be mixed within the same area. This is fundamental for developing innovative drug testing and screening platforms (Park et al., 2018b). By combining the electrical recording with the impedance measurements, it is possible to have a clear map of both the cellular morphology as well as of its electrophysiological fingerprint. Figure 6B,C demonstrate the recording of the electrical activity and the impedance imaging, respectively, in a selected area of the chip of a 43 DIV culture. As it can be appreciated from the pictures, areas with lower activity correspond to areas of lower impedance variation. This can be a fast and non-invasive tool to discriminate between areas of the chip which do not show any electrical activity, despite being covered by cells, from areas which do not present activity due to missing cell coverage or to non-uniform culture conditions. These results represent a preliminary proof-of-concept for our multi-modal CMOS MEA as an innovative platform not only to record the activity of electrogenic cells but also to monitor their spatial distribution with a label free, real time and non-invasive approach.

## Conclusion

In this work, we reported impedance imaging and voltage recording of primary rat hippocampal neuronal networks grown on a multimodal CMOS MEA chip. The multimodality of the chip allowed us to monitor simultaneously the electrophysiological activity and the growth of the primary neurons *in vitro*. The measurements derived from impedance monitoring at 1 kHz allowed us to discern, with only electrical imaging, between a freshly seeded (4.5 h after seeding) and a confluent (8 DIV) culture. Therefore, electrical imaging allows for a potential microscope-free non-invasive and fast technique to monitor cell cultures at different development stages. Further, as opposed to regular staining and microscopy it does not require end point measurements, sample preparation nor trained personnel for the manipulation. Numerous episodes of synchronized activity were observed and recorded. The maximum SNR was on average 10.1 ± 1.9 dB, therefore assuring a clear distinction between the measured signals and noise. An electrode-size dependency was observed for both the SNR, the noise and the maximum amplitude recorded on the different electrode groups present on the chip. By reducing the electrode area, the maximum SNR recorded on PHN neuronal cultures with our planar electrodes does not degrade significantly. In contrast, a higher maximum spike amplitude was detected by smaller electrodes if compared with larger ones. This is related to reduction of the signal amplitude due to spatial-averaging effects across the recording area of the electrodes. The high electrode density enabled to monitor the culture over four active areas (each 500 × 500 μm^2^) and 4096 electrodes, accessing 1024 at a time. Due to the flexibility in the modality selection and to the high electrode density on the CMOS MEA, it will be possible to further optimize both the impedance and voltage recording layouts. In this way, a larger spatial range can be covered addressing multiple sites simultaneously (e.g., to study cell culture heterogeneity) or specific focus can be given to a target area according to the final application. Additionally, the other four modalities (intracellular recording, current and voltage stimulation and impedance spectroscopy) can be applied in the same experiment, which further widens the potential applications of the presented platform. The different electrode sizes can also be exploited, together with the chip versatility, to assess physiological phenomena happening at very different scales e.g., ranging from axonal propagation up to network dynamics. Therefore, it is evident how multi-modality is a key element for the development of cutting edge MEA platforms. Finally, by being able to employ electrical imaging at different stages of the cellular growth *in vitro*, without interfering with the cellular growth, it is possible to perform simultaneous assessment of different physiological properties of the cultured neurons. Therefore, this tool can pave the way both to answer complex fundamental neuroscience questions as well as to aid the current drug-development paradigm.

## Ethics Statement

All the experiments involving live animals were executed according to the guidelines approved by the local university animal ethics committee and compliant with the European Communities Council Directive of November 24, 1986 (86/609/EEC).

## Author Contributions

BM conceived and planned the experiments with the support of DB and CL and conducted the experiments and analyzed the data. BM, CL, EG, and DB contributed to the manuscript preparation, results interpretation, and provided critical feedback. CL contributed to the chip design. JP developed the data acquisition software with the support of MS. OK worked on the primary cell cultures with the support of BM, helped during the fluorescent imaging, and obtained the SEM characterizations. S-WC helped in the data analysis of neuronal recordings. AF and AA worked on the device fabrication and postprocessing. VR and DB supervised and managed the study.

## Conflict of Interest Statement

The authors declare that the research was conducted in the absence of any commercial or financial relationships that could be construed as a potential conflict of interest.
