# Supplementary figures and images for: High-Density Electrical Recording and Impedance Imaging With a Multi-Modal CMOS Multi-Electrode Array Chip
Source: Front Neurosci. 2019 Jun 25;13:641. doi: 10.3389/fnins.2019.00641 (PMC6603149; doi:10.3389/fnins.2019.00641)

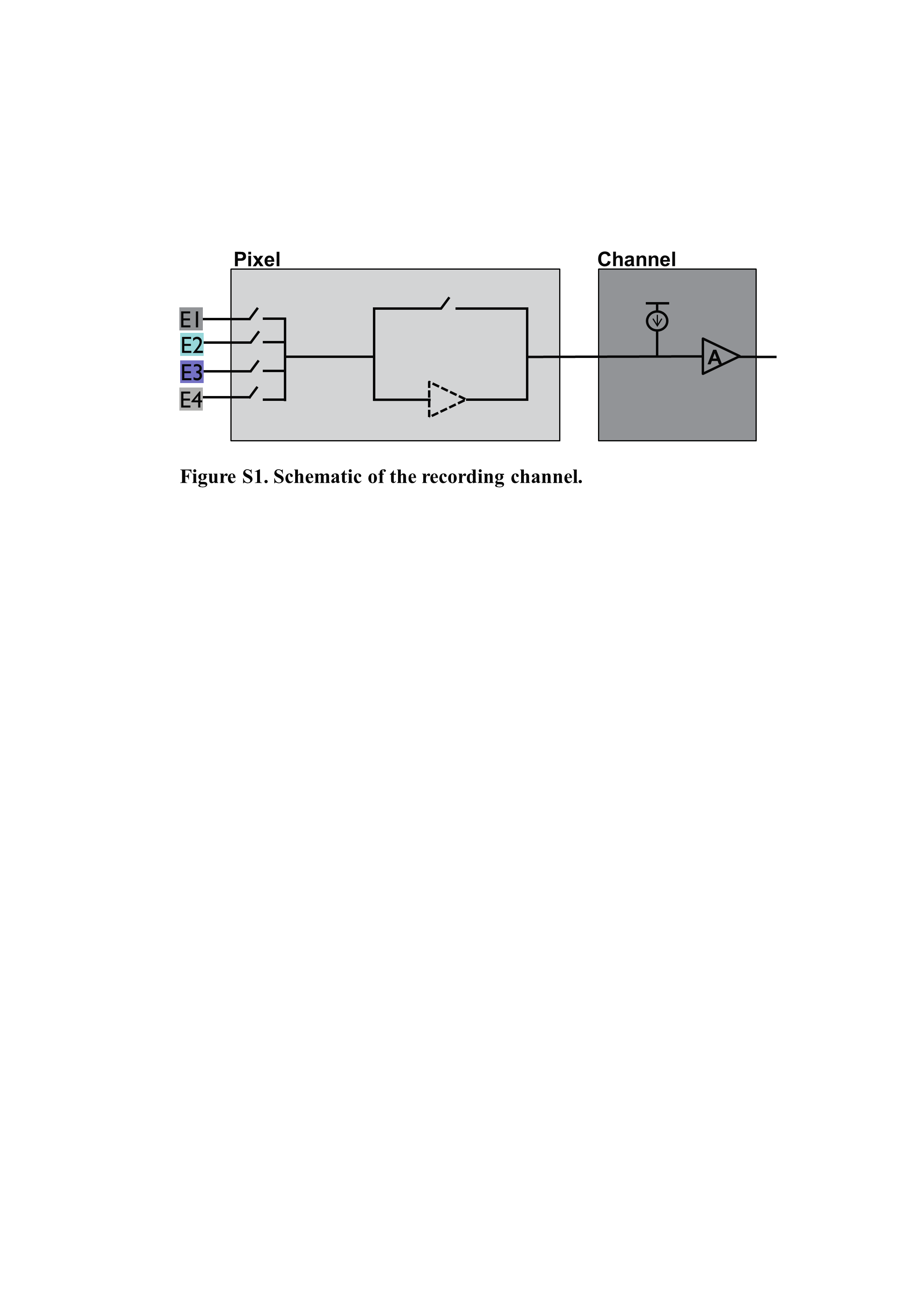

Supplement: Supplementary file 1 [file Image_1.tif]

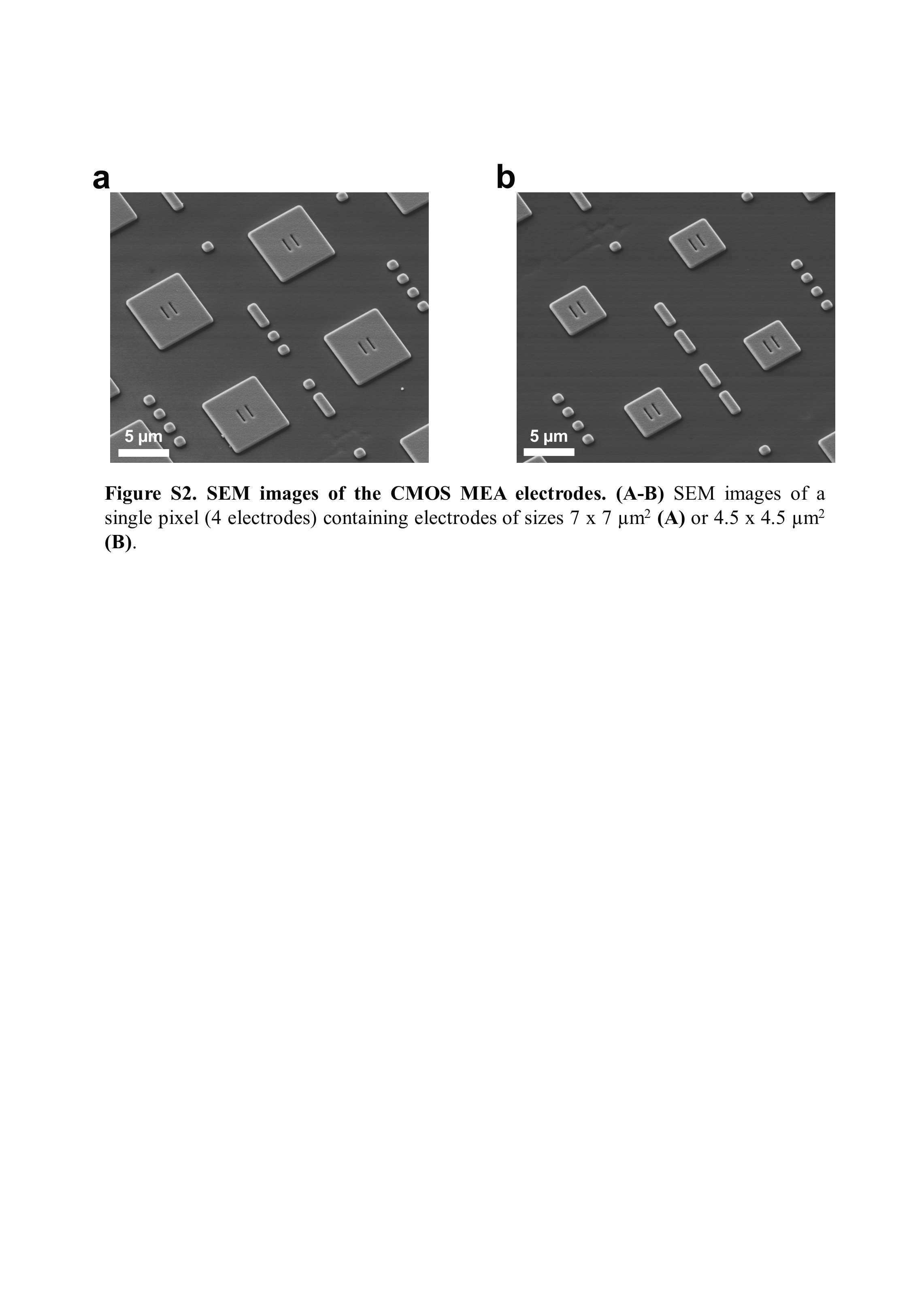

Supplement: Supplementary file 2 [file Image_2.tif]

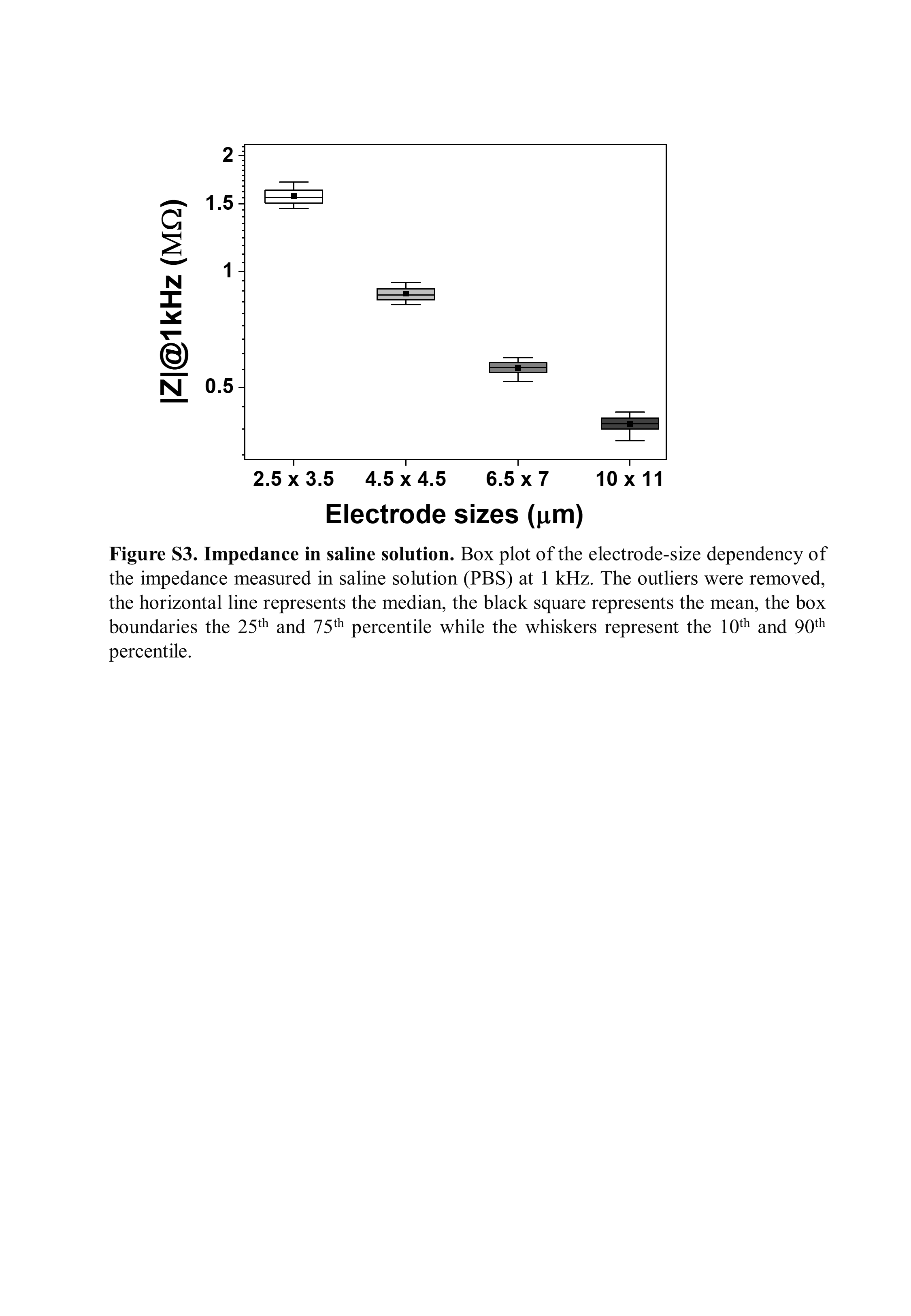

Supplement: Supplementary file 3 [file Image_3.tif]
